# On-Demand Breaking of Action-Reaction Reciprocity between Magnetic Microdisks Using Global Stimuli

**DOI:** 10.1103/PhysRevLett.131.058301

**Published:** 2023-08-04

**Authors:** Gaurav Gardi, Metin Sitti

**Affiliations:** 1 Physical Intelligence Department, Max Planck Institute for Intelligent Systems, 70569 Stuttgart, Germany; 2 Department of Physics, University of Stuttgart, 70569 Stuttgart, Germany; 3 School of Medicine and College of Engineering, Koç University, 34450 Istanbul, Turkey; 4 Institute for Biomedical Engineering, ETH Zurich, 8092 Zurich, Switzerland

## Abstract

Coupled physical interactions induce emergent collective behaviors of many interacting objects. Nonreciprocity in the interactions generates unexpected behaviors. There is a lack of experimental model system that switches between the reciprocal and nonreciprocal regime on demand. Here, we study a system of magnetic microdisks that breaks action-reaction reciprocity via fluid-mediated hydrodynamic interactions, on demand. Via experiments and simulations, we demonstrate that nonreciprocal interactions generate self-propulsion-like behaviors of a pair of disks; group separation in collective of magnetically nonidentical disks; and decouples a part of the group from the rest. Our results could help in developing controllable microrobot collectives. Our approach highlights the effect of global stimuli in generating nonreciprocal interactions.

## Introduction

Reciprocity, every action is faced with an equal and opposite reaction, is prevalent under equilibrium conditions. However, nonreciprocity in interactions is common in systems out of equilibrium and gives rise to unique behaviors that are not exhibited by the equilibrium systems [1–10]. Biological entities [11–13] typically operate out of equilibrium and they break action-reaction symmetry [14] for various purposes, such as locomotion and self-organization [1,2]. Investigating the role of nonreciprocity in the behavior of nonequilibrium systems would allow us to expand our understanding of various complex biological systems. This motivation has led to a variety of theoretical predictions regarding the behaviors that nonreciprocal interactions can produce [3–7], including translation of two identical objects rotating in opposite directions [15,16]. To reinforce our understanding of such effects, these theoretical predictions need to be complemented with their experimental realizations. Existing systems include robotic platforms [3,17] that are governed by computer algorithms, akin to computer simulations. However, to test the theoretical predictions under the influence of coupled physical interactions, there is a need to develop a versatile experimental system that is powered by physical interactions and that can break action-reaction reciprocity on demand. Apart from providing fundamental insights into the effect of nonreciprocity, such an experimental system could also potentially guide the development of intelligent and controllable machines at the microscopic scales that are useful for various biomedical and environmental applications [18,19].

In this quest for a versatile experimental model system, many nonequilibrium systems have been developed, ranging from active colloids [20–27] to global stimuli-driven collectives [28–37]. Similar to the biological entities, the constituents of active systems can individually harness available energy via different mechanisms to exhibit self-propulsion. However, it is usually difficult to control the behavior of active systems and the action-reaction symmetry is always broken in such systems. In contrast to active systems, the behavior of individual constituents of an externally driven system is usually monotonous: they follow the external stimuli exactly. Nevertheless, the advantage of externally driven collectives is that they are often easier to control than the active self-propelled systems. While not yet available, an experimental collective system that can combine the advantages of both self-propelled and externally driven systems would be a versatile model system. In this study, we propose a solution to the challenge of adding programmable nonreciprocity in an experimental externally (globally) driven collective system by breaking the action-reaction reciprocity.

To this end, various systems composed of floating magnetic spheres have been proposed [38,39]. However, these systems either require an external confinement or a physical connection between two spheres to maintain a stable configuration. An ideal model system would be one in which the physical interactions between the constituents can be tuned via global stimuli to maintain a stable configuration. Recently, magnetic microdisk collectives [40] have been shown to exhibit more diverse behaviors than any other externally driven system at the submillimeter scale, and the tunability of the pairwise interactions in the system using a global magnetic field makes it suitable for this study. Here we demonstrate two methods by which action-reaction reciprocity can be broken (on-demand) in the hydrodynamic interactions between a pair of magnetic microdisks floating at the air-water interface. The first method exploits a one-dimensional (1D) oscillating magnetic field to generate nonreciprocal interactions among a pair of identical microdisks and the second method relies on the difference in the magnetic response of a pair of magnetically nonidentical microdisks. In both cases, the pair propels as a single unit, like a self-propelling active particle. Finally, nonreciprocal interactions produce group separation in a collective composed of two types of disks, allowing a part of the collective to be decoupled from the rest. Our findings could be extended to other collective systems composed of hydrodynamically interacting agents.

## Results

Collective magnetic microdisk system: Each magnetic disk is 150 μm in radius (*R*) with symmetrically placed cosine profiles at their edges to generate pairwise capillary interactions (see Supplemental Material [41] for more details). The disks are coated with cobalt (Co) to generate a permanent in-plane magnetic dipole moment [41]. A spatially uniform external magnetic field is used to exert torques on the disks and induce their oscillation about their individual c.m. The torque on the ith disk due to the external magnetic field can be written as

(1) $$T_{i}=m_{i}B_{0}\sin\left(\theta -\alpha_{i}\right),$$

where *m_i_* is the magnetic moment and *α_i_* is the orientation of the *i*th disk, and *B*_0_ is the magnitude and *θ* is the orientation of the external magnetic field.

A pair of disks interact with each other via three pairwise interactions: capillary interactions [42,43] (induced by the cosine profiles), magnetic dipole-dipole interactions (induced by the deposited cobalt layer on the disks), and hydrodynamic lift force [28] (induced by the instantaneous angular velocities of the disks). The capillary interactions and magnetic dipole-dipole interactions are dependent on the relative orientation of the disks and are repulsive and attractive, respectively, when averaged over one full rotation of the disks. The hydrodynamic lift force depends on the instantaneous angular velocities of the disks and is always repulsive. The balance between the attractive and repulsive interactions enables the disks to maintain a finite steady-state distance (~*R*) while spinning about their individual centers. The disks also generate an azimuthal flow field due to angular displacement about their individual centers. This azimuthal flow generates pairwise interactions in the transverse direction to the center-center axis of a pair of disks. This transverse interaction manifests as transverse velocity of the disks. The hydrodynamic lift force and the transverse velocity caused by the jth disk on the ith disk can be written as

(2) $$F_{\text{hydro}\;}^{ij}=\frac{\rho R_{i}^{4}R_{j}^{3}\omega_{j}^{2}}{d^{3}},$$

(3) $$v_{\text{transverse}\;}^{ij}=\frac{R_{j}^{3}\omega_{j}}{d^{2}},$$

where *ρ* is the density of water, *R_i_* and *R_j_* are the radius of *i*th and *j*th disk, *ω_j_* is the instantaneous angular velocity of the *j*th disk, and *d* is the distance between the two disks (see Supplemental Material [41] for the pairwise interaction model). All the disks used in this study have the same radius (*R_i_* = *R_j_* = 150 μm, ∀ *i*, *j*).

**FIG. 1:**
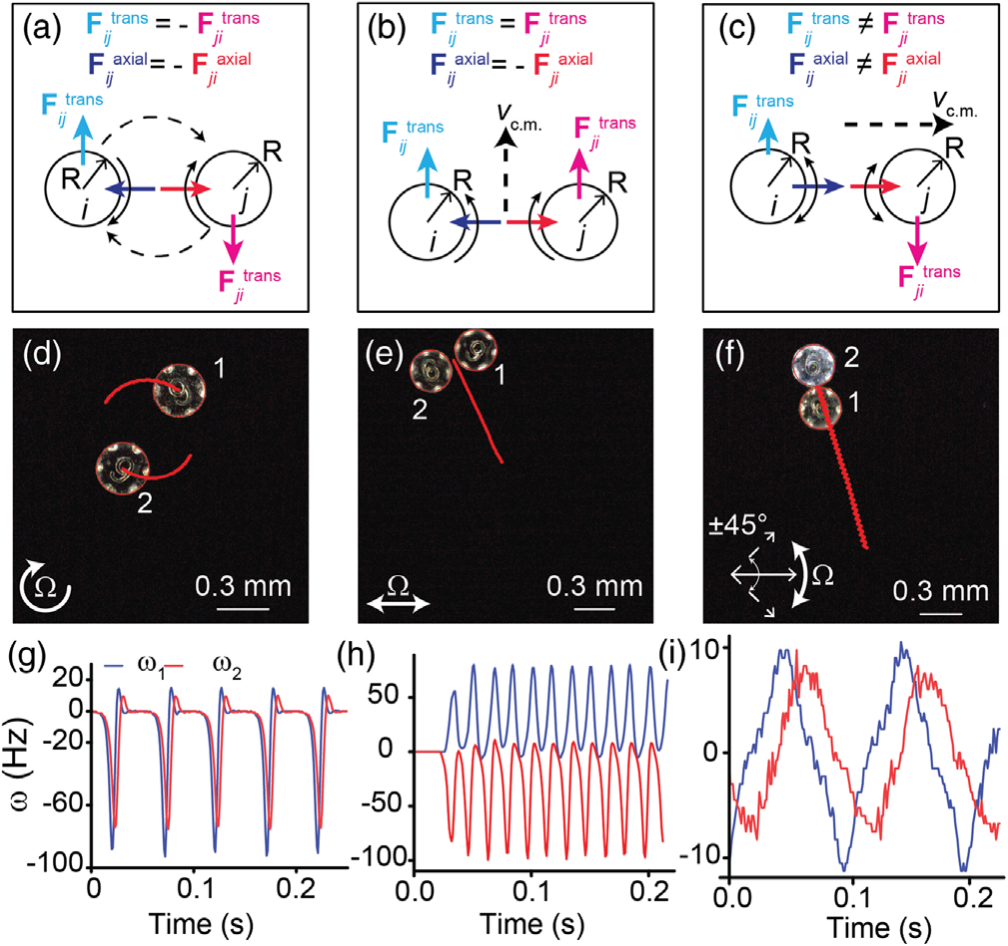
Different scenarios of pairwise interactions of two magnetic microdisks on the air-water interface. (a)−(c) Schematic of the cases of reciprocal and nonreciprocal pairwise interactions between two magnetic microdisks. Solid black arrows represent the spinning directions of the disks and the dashed black arrows represent the direction of orbiting of the disks in (a) and the direction of translation of the c.m. in (b) and (c). (d)–(f) Experimental images showing the behavior of a pair of microdisks corresponding to the cases illustrated in (a)-(c).The golden microdisks have higher magnetic moment than the gray one. The red lines show the trajectory of the disks in (d) and of the c.m. in (e) and (f). See Supplemental Material [41] for the details on the fabrication of the disks. (g)–(i) Angular velocities of the two disks corresponding to the experiments in (d)–(f).

The angular velocity of the disks can be controlled using the external magnetic field. As evident from Eqs. (2) and (3), two disks having opposite instantaneous angular velocities (*ω_i_* = −*ω_j_*) or different angular speeds (*ω_i_* ≠ *ω_j_*) interact with each other nonreciprocally $[F_{\text{hydro}\;}^{ij}(d)\neq F_{\text{hydro}\;}^{ji}(d)$ or $v_{\text{transverse}\;}^{ij}\neq v_{\text{transverse}\;}^{ji}].\;$. The behavior of a pair of disks for different scenarios of reciprocal and nonreciprocal regime are shown in Fig. 1. When a pair of identical disks spins with the same angular velocities (*ω_i_* = *ω_j_*), they maintain a steady-state distance while orbiting around a common center of mass [Figs. 1(a), 1(d), and 1(g)]. Such a behavior in a reciprocal regime can be achieved by using a rotating magnetic field **B**, where

(4) $$B=B_{0}\cos\text{Ω}t\cdot \hat{x}+B_{0}\sin\text{Ω}t\cdot \hat{y}.$$

Here, *B*_0_ is the magnitude and Ω is the angular velocity of the external magnetic field, and *t* is time.

Inducing nonreciprocity along the transverse direction: The reciprocity in transverse interactions can be broken for a pair of disks spinning in opposite directions (*ω_i_* = −*ω_j_*) as described by Eq. (3). The experimental realization of the theoretically predicted translation of identical rotors [15,16] can be achieved using a 1D oscillating magnetic field [Figs. 1(b), 1(e), and 1(h)], where

(5) $$B=B_{0}\cos\text{Ω}t\cdot \hat{x}.$$

**FIG. 2:**
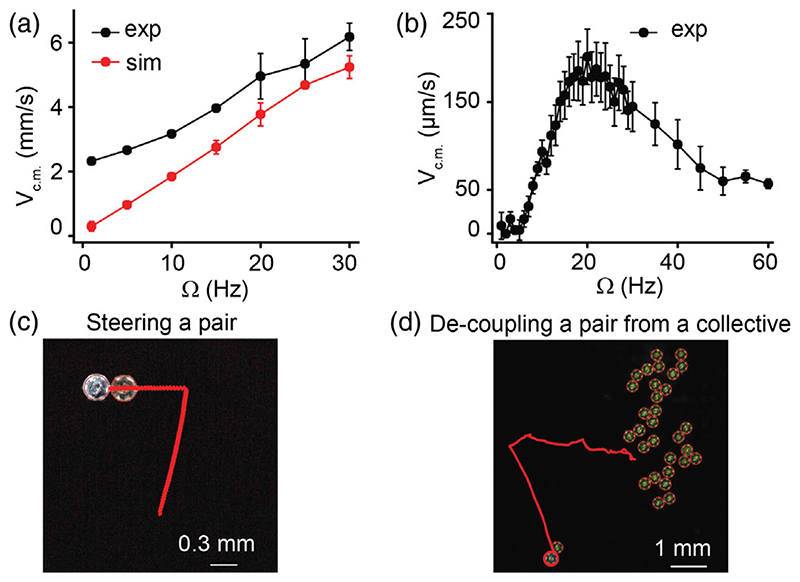
Translation speeds, steering, and decoupling a pair from a collective. (a) Translation speed of the c.m. (*V*_c.m._) vs frequency of external magnetic field Ω for the counterrotating disks. Black and red curves correspond to experiments and simulations, respectively. (b) *V*_c.m._ vs Ω for a pair of magnetically nonidentical disks in experiments. (c) A pair of magnetically nonidentical disks can be steered by changing the mean axis of oscillation of the external magnetic field. (d) A collective containing 31 microdisks with a stronger magnetic moment (golden) and one disk having a weaker magnetic moment (gray). The pair containing the gray and one golden disk starts translating and the direction of translation can be steered without affecting the remaining disks. The red lines in (c) and (d) represent the trajectory of the c.m.

Because the magnetic dipole on the microdisks tends to align with the external magnetic field, when the direction of the magnetic field switches it creates an unstable state (due to 180° angle between the magnetic dipole on the disk and the external magnetic field vector). Therefore, a disk can rotate either clockwise or counter-clockwise to align with the external magnetic field, generating a pair of disks with opposite instantaneous angular velocities. Each microdisk in the pair now exerts the transverse interaction in the same direction as the other, resulting in a translation of the pair (Supplemental Material [41], movies S1 and S2). Two counterrotating disks translate [Figs. 1(e) and 1(h)], and co-rotating disks orbit around a common center of mass (c.m.) similar to the case of rotating magnetic field [Figs. 1(d) and 1(g)]. The translation velocity of c.m. as a function of time also shows an oscillatory behavior (Fig. S2 [41]) similar to the angular velocities of the disks, as expected from Eq. (3), because the torque due to the external magnetic field is dependent on the angle the magnetic dipole makes with the external magnetic field [Eq. (1)]. The translation speed of the c.m. increases with Ω and the trend is captured in the numerical simulations [Fig. 2(a) and movie S1 of [41]]. The pairwise interactions model is described in the Supplemental Material [41].

The disks tend to maintain their direction of rotation unless an external perturbation (like collision with the physical boundary) disturbs their motion. This could be due to fluidic torque at finite Reynolds number (~10^−3^ ™ 10) and the orientation-dependent capillary torques (see pair formation section in the Supplemental Material [41]). Occasionally, one of the disks changes its direction of rotation and the disks momentarily orbit around their c.m., changing the direction of propulsion of the pair (movie S1 [41]).

Inducing nonreciprocity along the axial direction: The second case of nonreciprocity [Fig. 1(c)] is achieved by using a pair of disks that are identical in geometry but differ in the strength of their magnetic dipole moment *m* [Fig. 1(f)]. The torque applied by the external magnetic field on a microdisk depends on the disk’s m. For the same lag (*θ* ™ *α*), a disk with smaller *m* experiences weaker torque due to the external magnetic field than a disk with higher value of *m* does [Eq. (1)]. Moreover, when the external magnetic torque is smaller than the rotational viscous drag (for Ω >step-out frequency), a disk cannot synchronously follow the external magnetic field. Intuitively, the lower the magnetic dipole moment of the disk, the lower the step-out frequency. Additionally, a disk with lower *m* tends to lag further behind the external magnetic field vector than a disk with higher *m*.

We use two types of disks, each with a different value of *m* (see Supplemental Material [41] for fabrication). For clarity, we refer to a disk with higher *m* as type-1 and a disk with lower *m* as type-2 disk, respectively. Next, we use a magnetic field oscillating in two dimensions (2D) about a mean-axis:

(6) $$B=B_{0}\cdot \hat{x}+B_{0}\sin\text{Ω}t\cdot \hat{y}.$$

In the experiments, we observe that the pair translates along the axial direction with the type-2 disk leading and the type-1 disk trailing [Fig. 1(f) and movie S3 [41]] and the translation speed of the c.m. depends on Ω [Fig. 2(b)]. A closer look at the instantaneous angular velocities of the disks shows a lag between the two angular velocities that can be attributed to the difference in their magnetic moments [Fig. 1(i)]. Interestingly, we observe that the direction of propulsion of the disks can be changed by changing the mean-axis of the oscillation of the external magnetic field [Fig. 2(c) and movie S4 [41]].

When a type-2 disk is inserted within a collective containing multiple type-1 disks, the type-2 disk can selectively attach to a single type-1 disk and decouple it from the remaining disks. The pair of type1-type2 disks can be manipulated to move around while the rest of the collective stays stationary on average [Fig. 2(d) and movie S5 [41]]. This is an example of a system where majority of the constituents are interacting reciprocally while only a pair interacts nonreciprocally. By increasing the number of type-2 disks we can study how the nonreciprocity affects the behavior of a heterogeneous collective.

**FIG. 3:**
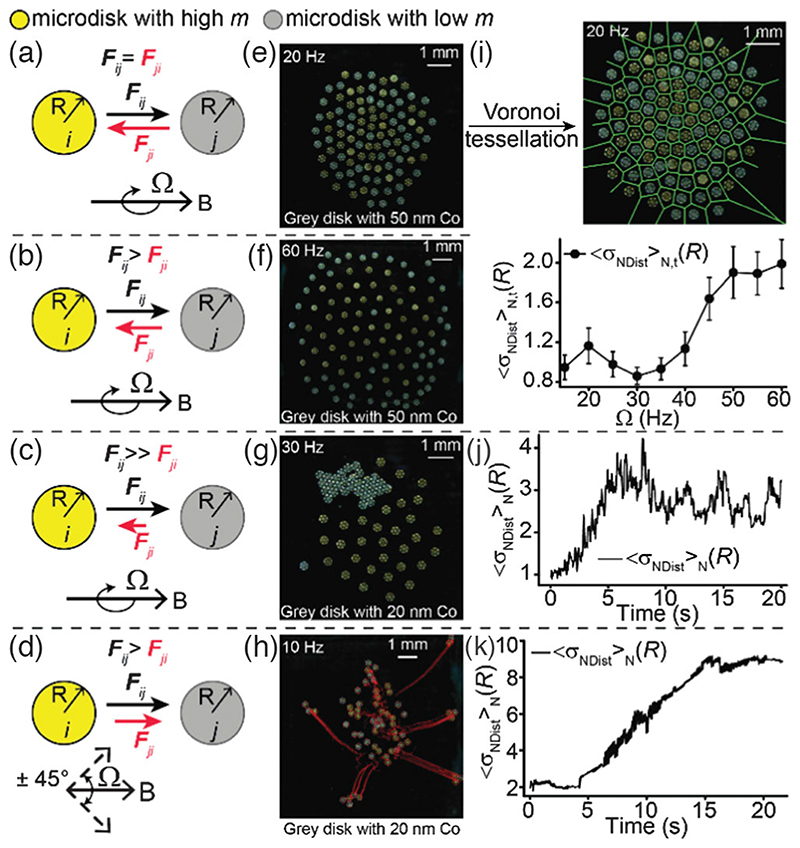
Behavior of many disks with identical geometry and different magnetic moments. (a)−(d) Schematics of the pairwise interactions. (e)–(g) Experimental behavior of the collective under a rotating magnetic field at Ω = 20 (e), Ω = 60 (f), and Ω = 30 Hz (g). The microdisks with low *m* (gray) step out in (f) and (g) and are pushed to the periphery. The gray disks in (g) have lower *m* than those in (e) and (f) and step out below 25 Hz. (h) The collective splits into stationary and translating groups (trajectories represented by red lines) under an oscillating magnetic field [Eq. (6)]. (i) Example of the Voronoi tessellation (top) and standard deviation of neighbor distances as a function of Ω (bottom). Neighbors are identified using the Voronoi tessellation. (j)–(k) Standard deviation of neighbor distances as a function of time for the experiments shown in (g)–(h).

Collective behavior of group of type-1 and type-2 disks: We study the behavior of a collective consisting of equal numbers of type-1 and -2 disks (Fig. 3). In the reciprocal regime [Fig. 3(a)], for a rotating magnetic field [Eq. (4)], the collective behaves like a homogeneous system where each microdisk behaves almost identically to each other [Fig. 3(e) and movie S6 [41]]. On increasing Ω above the step-out frequency of type-2 disks, the type-2 microdisks start to step out and spin at lower angular velocities than those of type-1 disks, causing nonreciprocal interactions between type-1 and type-2 disks [Figs. 3(b) and 3(c)]. A type-1 disk repels a type-2 disk stronger and also causes higher transverse velocities of type-2 disks than vice versa. Consequently, the type-2 disks tend to be pushed to the boundary of the collective where the length of the circumference is larger, allowing the type-2 disks to orbit faster about the collective’s center of mass and resulting in the separation of the two disk types [Fig. 3(f) and movie S7 [41]]. We use standard deviation of neighbor distance distribution as an order parameter to quantify the extent of separation of the disks [Fig. 3(i)]. The neighbors are identified using Voronoi tessellation [44]. This separation appears clearer for the type-2 disks with even smaller *m*, where the type-2 disks are pushed out before assembling into a tiled structure while the type-1 disks still form a rotating collective [Figs. 3(g) and 3(j), and movie S8 [41]]. This transition from a homogeneous to a separated behavior is a consequence of breaking the action-reaction reciprocity on demand.

Finally, an external magnetic field described by Eq. (6) causes separation of the group into smaller groups that each contain several microdisks. The groups containing unequal numbers of type-1 and type-2 disks start to translate while the remaining disks stay stationary on average [Fig. 3(h) and movie S9 [41]]. This separation is also indicated by the increase in the standard deviation of neighbor distances [Fig. 3(k)]. Finally, Fig. S3 shows the tiling process of the collective containing equal numbers of type-1 and type-2 disks (movie S10 [41]). This experiment is similar to those in previous studies (experimental protocol in Ref. [41]) and it shows that in the reciprocal regime the collective of nonidentical microdisks can form an ordered structure just like a collective containing identical disks [44].

## Discussion

In this study, we present two methods to experimentally break action-reaction reciprocity among hydrodynamically interacting objects like the magnetic microdisks. We demonstrate various pairwise and collective behaviors generated by the nonreciprocal interactions.

First, we present an experimental realization of theoretically predicted behaviors of two identical rotors [15,16]. We utilize an external magnetic field oscillating in 1D to realize two counter-rotating disks that maintain a steady-state distance while translating at speeds of about a few mm/s (tens of body length/s). The second method relies on a nonidentical magnetic response of microdisks, where an external magnetic field oscillating in 2D affects the different magnetic moments. The direction of translation of the pair can be tuned by changing the mean-axis of oscillation of the external magnetic field. Next, we show that a single microdisk with a weaker magnetic moment can attach to any disk with a stronger magnetic moment, and the pair can be decoupled from the rest of the collective. Finally, transitioning to the nonreciprocal regime, a heterogeneous collective separates into groups of disks with strong and weak magnetic moments, allowing a part of the collective to be decoupled from the rest.

The ability of transitioning from reciprocal to nonreciprocal regime using 1D magnetic field enables our system to be useful for testing theoretical insights on the collective behavior of a group of co-rotating and counter-rotating particles [6,7]. Moreover, the ability to tune the steady-state distance between the disks (Fig. S4 [41]), without requiring any external confinement or physical connection between the disks, makes our system suitable for studying collective behaviors that emerge from the nonreciprocal interactions. Our findings can also be useful for the development of collective microrobots for various biomedical and environmental applications [18,19]. Experimental techniques that can decouple a part of the collective from the rest [Figs. 2(d), 3, movies S5, S7-9, and S11 [41]) can potentially augment the existing computation techniques [45] to generate microrobot collectives that are more controllable and thus able to perform multiple tasks in parallel.

While we present many behaviors induced by the nonreciprocity, much more remains to be explored. For example, the tiling process of the disks [41] can be compared to the recently studied living chiral crystals [1], and the theoretical insights on odd elasticity [5] can be tested for the experiments shown in Fig. 3(g) and movie S8, where a part of the collective exhibits a tiled structure and the rest forms a rotating group. Moreover, the similarity of our system’s behavior to that of the predator-prey-like droplet pairs [20] that interact via chemical signals provides an opportunity to connect the complex chemical signal-induced interactions to the better-understood physical interactions like magnetic dipole-dipole interactions and hydrodynamic interactions. Our system’s versatility and adaptability enable a model collective system for various fundamental and robotic studies.

The authors thank C. Holm, C. Lohrmann, and M. Zhang for discussions, N. K.-Subbaiah for discussions about two-photon polymerization-based 3D microprinting of the disks, and sputtering process, and A. Shiva for help on measurement of magnetic hysteresis curves and discussions on sputtering process, and D. Sheehan for proofreading the text. The authors thank the Max Planck Society for funding. G. G. thanks the International Max Planck Research School for Intelligent Systems (IMPRS-IS) for support.

## Supplementary Material

SI

Video 1

Video 3

Video 2

Video 4

Video 5

Video 6

Video 7

Video 8

Video 9

Video 10
