# Supplementary material for: On-Demand Breaking of Action-Reaction Reciprocity between Magnetic Microdisks Using Global Stimuli
Source: Phys Rev Lett. Author manuscript; Available in PMC 2023 Sep 26. (PMC7615123; doi:10.1103/PhysRevLett.131.058301)
Supplement: SI [file EMS185224-supplement-SI.pdf]

Supplemental information for

On demand breaking of action-reaction reciprocity between magnetic micro-disks using global stimuli

Gaurav Gardi[1,2], Metin Sitti[1,3,4*]

[1] Physical Intelligence Department, Max Planck Institute for Intelligent Systems, 70569 Stuttgart, Germany.
[2] Department of Physics, University of Stuttgart, 70569 Stuttgart, Germany
[3] School of Medicine and College of Engineering, Koç University, 34450 Istanbul, Turkey
[4] Institute for Biomedical Engineering, ETH Zurich, 8092 Zurich, Switzerland

* Corresponding author: Metin Sitti
Email: sitti@is.mpg.de

This file includes:

Supplemental information on

- Fabrication of micro-disks
- Video acquisition
- Experimental protocols
- Pair formation
- Calculation of the distribution of the neighbor distances
- Pairwise interaction model

Figures S1 to S6
Description of Movies S1 to S11
References

Other supplemental materials for this manuscript include the following:

Movies S1 to S11

Fabrication of micro-disks

The design of micro-disks was generated in Rhinoceros 3D using the Grasshopper plug-in. The generated STL file was used to fabricate the disks using two photon polymerization (Nanoscribe Photonic Professional GT). A 25x objective was used to write the designed micro-disks within IP-S photoresist in the dip-in mode. The following writing parameters were used: 1) Adaptive slicing distance: minimum of 0.5 µm and a maximum of 3 µm; 2) Hatching distance: 0.3 µm; 3) Hatching angle: 45°; 4) Hatching angle offset: 72°; 5) Number of contours: 3.

Thin films of cobalt, gold and platinum were sputtered onto the micro-disks using a sputter coater (Leica EM ACE600). The base vacuum pressure before the sputtering was $<5 \times 10^{-6}$ mbar. Cobalt was sputtered using 100 mA current at a sputtering pressure of 2×10^{-2} mbar. A pre-sputtering process was used for 60 s to remove the oxide layer on the Cobalt target. Gold was sputtered using 30 mA current at a sputtering pressure of 8×10^{-3} mbar. Platinum layer was sputtered using 35 mA current at a sputtering pressure of 8×10^{-3} mbar. The platinum and gold layers act as passivating layer and protect the cobalt layer from getting oxidized. The sputtering procedure could be finished within a few hours.

The 3D structure of the printed disks was observed using a laser scanning confocal microscope (Keyence VK-X200 series) with a 20x objective. The magnetic hysteresis was measured for a 10 mm x 10 mm piece of glass using a vibrating sample magnetometer (VSM, MicroSense EZ9). The magnetic moment measured for a 10 mm x 10 mm glass piece was scaled down to get the values for a disk having 300 μm diameter for use in the numerical simulations.

Video acquisition

A Basler acA2500-60uc was used to record experimental videos involving many disks and Phantom Miro Lab140 was used to record videos at high frame rate (1000 frames per

second (fps)) of the experiments involving a pair of disks. The experiments were performed inside the field of view of a Leica manual zoom microscope Z16 APO and the cameras were mounted on the microscope to record the videos of the experiments. Illumination was provided by a LED light source (SugarCUBE Ultra) connected to a ring light guide (0.83'' ID, Edmund Optics #54-176).

A custom python code using the OpenCV library was written to analyze the experimental videos. The detected data was used to calculate the orientations, angular speeds and hexatic order parameter of the disks, and the speed of the center of mass (COM) of the disks. Another custom-made python script was used to overlay the raw images with trajectories of the disks or their COM.

Experimental protocols

All the experiments were performed in a square boundary with 10 mm side. The amplitude of the external magnetic field was 10 mT (B_0) for all the experiments. Experiments shown in Fig. 1e were performed using micro-disks having a 500 nm-thick cobalt and 60 nm-thick gold film (type 1). The frequency of the 1D oscillating magnetic field was 30 Hz. The videos were recorded at a framerate of 1000 fps.

Experiments shown in Fig. 1f were performed using micro-disks having different magnetic dipole moments. The type-1 (golden) disks were coated with a 500 nm-thick cobalt and 60 nm-thick gold film and the type-2 (grey) disks were coated with a 20 nm-thick cobalt, 60 nm-thick gold and 20 nm-thick platinum film. A layer of platinum was coated to visually distinguish the type-1 and type-2 disks. The frequency of the 2D oscillating magnetic field was 10 Hz. The experiments in Fig. 2c were performed using 31 disks of type-1 and one disk of type-2. The videos were recorded at a framerate of 1000 fps. It should be noted that the direction of translation of the pair could be either along the

positive or the negative direction of the mean axis of oscillation of the external magnetic field (depending on the initial orientation of the pair).

Experiments shown in Fig. 3e and f were performed using 50 type 1 and 50 type 2 disks. The type 1 (golden) disks were coated with 500 nm cobalt and 60 nm gold and the type 2 (grey) disks were coated with 50 nm cobalt, 60 nm gold and 20 nm Platinum. A layer of platinum was coated to visually distinguish the type 1 and type 2 disks. Experiments in Fig. 3g and h were performed using 31 disks of type 1 and 31 disks of type 2. The type 2 disks in this case were coated with 20 nm-thick cobalt, 60 nm-thick gold and 20 nm-thick platinum. The frequency of the rotating magnetic field was 20 Hz (Fig. 3e), 60 Hz (Fig.3f) or 30 Hz (Fig.3g). The frequency of the 2D oscillating magnetic field was 10 Hz (Fig.3h). The videos were recorded at a framerate of 30 fps. The step-out frequency of type 2 disks having a 20 nm-thick cobalt layer was around 25 Hz and that of the type 2 disks having 50 nm-thick cobalt layer was around 50 Hz.

The frequency of the rotating magnetic field in the tiling experiments (Fig.S3) was 15 rotations per second (rps) for 5 s and then reduced to 1 rps for 15 s and to 0.25 Hz for 10 s. Finally, a mix of two different rotating magnetic fields (Ω = 1 Hz, B_0 = 3 mT for 1 s and Ω = 0.25 Hz, B_0 = 0.5 mT for 4 s) was used for 120 s to break and minimize the number of holes in the tiles to form a single continuous tile. The videos were recorded at 10 fps.

Pair formation

Under 1D magnetic field, a counter-rotating pair is slightly more likely to form because of two factors. First, a rotating disk also generates a weak fluidic torque on a neighboring disk and this torque tends to rotate the neighboring disk in the direction opposite to the original disk’s rotation direction (a clockwise rotating disk exerts a torque on a neighboring disk to rotate it in counter-clockwise direction and vice-versa). This fluidic torque is

normally weaker compared to the torque exerted by the external magnetic field on the disks and can be neglected. However, we hypothesize that due to the finite Reynolds number of our system ($\sim 10^{-3} - 10$), in case of 1D magnetic field (Eq. 5), the fluidic torque becomes comparable to the torque exerted by the external magnetic field (Eq. 1) when the magnetic field strength is close to zero ($\Omega t = \frac{\pi}{2}$ or $\Omega t = \frac{3\pi}{2}$ in Eq. 5) or when the magnetic dipole on the disks is almost aligned with the magnetic field ($\theta \simeq \alpha_i$ in Eq. 1). Therefore, the disks tend to keep rotating in their original direction of rotation. Second, we suspect that the orientation-dependent capillary torque also makes it harder for a disk to change its direction of rotation midway. While both these factors make it less likely for a disk to change its direction of rotation, occasionally we observe in experiments that one of the disks changes its direction of rotation while translating and the disks momentarily orbit around their center of mass, changing the direction of propulsion of the pair (Movie S1). In most of the scenarios, this switching appears to occur at random.

We also observed that in some cases both the disks rotate in the same direction. This scenario depends on the initial configuration of the disks. Empirically, we found that the initial configuration of the disks can be controlled by transitioning from an appropriate magnetic field profile to a 1D magnetic field (Eq. 5). For example, transitioning from the rotating magnetic field (Eq. 4) to a 1D field is more likely to result in a co-rotating pair. Transitioning from a magnetic field oscillating in 2D (Eq. 6) to a 1D magnetic field results in a counter-rotating pair.

Calculation of the standard deviation of the neighbor distances

A distribution of neighbor distances was calculated for each video frame corresponding to each experiment. The neighbors were identified using Voronoi tessellation. An example of Voronoi tessellation is shown in Fig. 3i (top). The standard deviation of neighbor distances was calculated for each video frame (in Fig. 3j-k) and the average of these

standard deviations over all the video frames was calculated for the steady-state behaviors in each experiment as shown in Fig. 3i (bottom). Standard deviations of neighbor distances increased with increasing Ω, opposite compared to the standard deviations of local density (Fig. S5).

Pairwise interaction model

The pairwise interaction model used for numerical simulations is presented below. This model was built on the previously reported model [1]. A Gaussian noise was added to the orientation of the disks for the case of 1D oscillating magnetic field. The mean and standard deviation of the noise was $0°$ and $10°$, respectively. The cosine profile at the edge of the disks deforms the air-water interface. This deformation causes capillary multipoles, resulting in orientation dependent capillary interactions. These interactions are same as those described in ref. [1]. These profiles may induce magnetic moment out of the plane, however, the behaviors of the disks can be replicated in simulations by assuming the out of plane components of magnetic dipoles to be negligible. Therefore, we assume that these out of plane components of the magnetic dipoles do not affect the interactions between the disks significantly to alter their behaviors that are studied in this work.

If the edge-edge distance *d* ≥ lubrication threshold (=15 μm, or 0.1R), then

$$\frac{d\boldsymbol{r_i}}{dt} = \sum_{j\neq i}(6\pi\mu R)^{-1}\left(F_{mag-on,\,i,j}(r_{ji},\varphi_{ji}) + F_{cap,\,i,j}(r_{ji},\varphi_{ji}\,) + \frac{\rho\omega_j^2 R^7}{r_{ji}^3}\right)\hat{r}_{ji} + \sum_{j\neq i}\left(\frac{F_{mag-off,\,i,j}(r_{ji},\varphi_{ji})}{6\pi\mu R} - \frac{R^3\omega_j}{r_{ji}^2}\right)\hat{r}_{ji}\times\hat{z},\ i = 1,2 \quad (1)$$

$$\frac{d\alpha_i}{dt} = \frac{m_i B\sin(\theta-\alpha_i)}{8\pi\mu R^3} + \sum_{j\neq i}\frac{T_{mag-\mathrm{d},i,j}(r_{ji},\ \varphi_{ji}) + T_{cap,i,j}(r_{ji},\ \varphi_{ji})}{8\pi\mu R^3},\ \ i = 1,2 \quad (2)$$

$$\alpha_i = \int\frac{d\alpha_i}{dt}\cdot d\tau + \eta_i(t) \quad (3)$$

$$\mathbf{B} = B_0\cos(\Omega_x t)\,\hat{\boldsymbol{x}} + B_o\sin(\Omega_y t)\,\hat{\boldsymbol{y}} \quad (4)$$

where $\boldsymbol{r_i}$ and $\boldsymbol{r_j}$ are the position vectors of micro-disks, $\boldsymbol{r_{ji}} = \boldsymbol{r_i} - \boldsymbol{r_j}$ is the vector pointing from the center of jth micro-disk to the center of ith micro-disk, α_i and α_j are the orientations of micro-disks, *d* is the edge-edge distance between two micro-disks,φ_{ji} is the angle of dipole moment on the micro-disks with respect to $\boldsymbol{r_{ji}}$, ω_j is the instantaneous angular speed of the jth micro-disk, Ω_x and Ω_y are the oscillation frequencies of the external magnetic field along x- and y- axis, respectively, $\theta = \arctan(\frac{\mathbf{B}\cdot\hat{\boldsymbol{x}}}{\mathbf{B}\cdot\hat{\boldsymbol{y}}})$ is the orientation of the magnetic field, R is the radius of the micro-disks and it is same for all the disks (150 µm), μ is the dynamic viscosity of water (10^{-3} Pa·s), ρ is the density of water (10^3 kg/m^3), m_i is the magnetic dipole moment of the ith micro-disk (10^{-8} A·m^2), $B = \left(B_0^2\left[\cos^2(\Omega_x t) + \sin^2(\Omega_y t)\right]\right)^{\frac{1}{2}}$ is the magnitude of the external magnetic field, $F_{mag-on,\,i,j}$ and $F_{mag-off,\,i,j}$

are the magnetic dipole-dipole force along the axial and transverse directions, respectively, $T_{mag-d,i,j}$ is the magnetic dipole-dipole torque and it depends on r_{ji} and φ_{ji} , $F_{cap,\,i,j}$ is the capillary force, and it depends on r_{ji} and φ_{ji} and embeds the symmetry of a micro-disk [1], and $T_{cap,i,j}$ is the capillary torque, which depends on r_{ji} and φ_{ji} and embeds the symmetry of a micro-disk, $\eta_i(t)$ is a gaussian noise with mean 0° and standard deviation $10°$. The capillary and magnetic interactions are defined according to the ref. [1].

If the edge-edge distance d < lubrication threshold (=15 µm, or 0.1R) and d ≥ 0, then

$$\mu \frac{d\boldsymbol{r_i}}{dt} = \sum_{j\neq i} A\left(\frac{d}{R}\right)\left(F_{mag-on,\,i,j}\left(r_{ji},\varphi_{ji}\right) + F_{cap,\,i,j}\left(r_{ji},\varphi_{ji}\ \right) + \frac{\rho\omega_j^2 R^7}{r_{ji}^3}\right)\hat{r}_{ji}$$
$$+\sum_{j\neq i} B\left(\frac{d}{R}\right) F_{mag-off,\,i,j}\left(r_{ji},\varphi_{ji}\right)\hat{r}_{ji}\times\hat{\text{z}} \qquad (6)$$
$$+\sum_{j\neq i} C\left(\frac{d}{R}\right) m_i B\sin(\theta-\alpha_i)\,\hat{r}_{ji}\times\hat{\text{z}},\ i=1{,}2$$

$$\mu \frac{d\alpha_i}{dt} = G\left(\frac{d}{R}\right) m_i B\sin(\theta-\alpha_i)$$
$$+\sum_{j\neq i} G\left(\frac{d}{R}\right) T_{mag-d,i,j}\left(r_{ji},\ \varphi_{ji}\right) + T_{cap,i,j}\left(r_{ji},\ \varphi_{ji}\right),\ i \qquad (7)$$
$$=1{,}2$$

where $A(x), B(x), C(x)$ and $A(x)$ the lubrication coefficients [1].

If the edge-edge distance d < 0, then a repulsion term is added to the force equation to prevent the overlap of any two micro-disks,

$$\mu \frac{d\boldsymbol{r_i}}{dt} = \sum_{j \neq i} A(\varepsilon) \left(F_{mag-on,\, i,j}\big(2R, \varphi_{ji}\big) + F_{cap,\, i,j}\big(2R, \varphi_{ji}\ \big) + \frac{\rho \omega_j^2 R^7}{r_{ji}^3} \right) \hat{r}_{ji}$$

$$+ \sum_{j \neq i} \frac{F_{wallRepulsion}}{6\pi R} \frac{-d}{R} \hat{r}_{ji}$$

$$+ \sum_{j \neq i} B(\varepsilon) F_{mag-off,\, i,j}\big(2R, \varphi_{ji}\big)\, \hat{r}_{ji} \times \hat{\mathrm{z}} \qquad (8)$$

$$+ \sum_{j \neq i} C(\varepsilon)\, m_i B \sin(\theta - \alpha_i)\, \hat{r}_{ji} \times \hat{\mathrm{z}},\ i = 1,2, \ldots$$

$$\mu \frac{d\alpha_i}{dt} = G(\varepsilon) m_i B \sin(\theta - \alpha_i)$$

$$+ \sum_{j \neq i} G(\varepsilon) T_{mag-d,i,j}\big(2R,\ \varphi_{ji}\big) + T_{cap,i,j}\big(2R,\ \varphi_{ji}\big),\ \ i \qquad (9)$$

$$= 1,2, \ldots$$

where ε is a small number (10^{-10} μm/R), and $F_{wallRepulsion}$ is set to be 10^{-7} N.

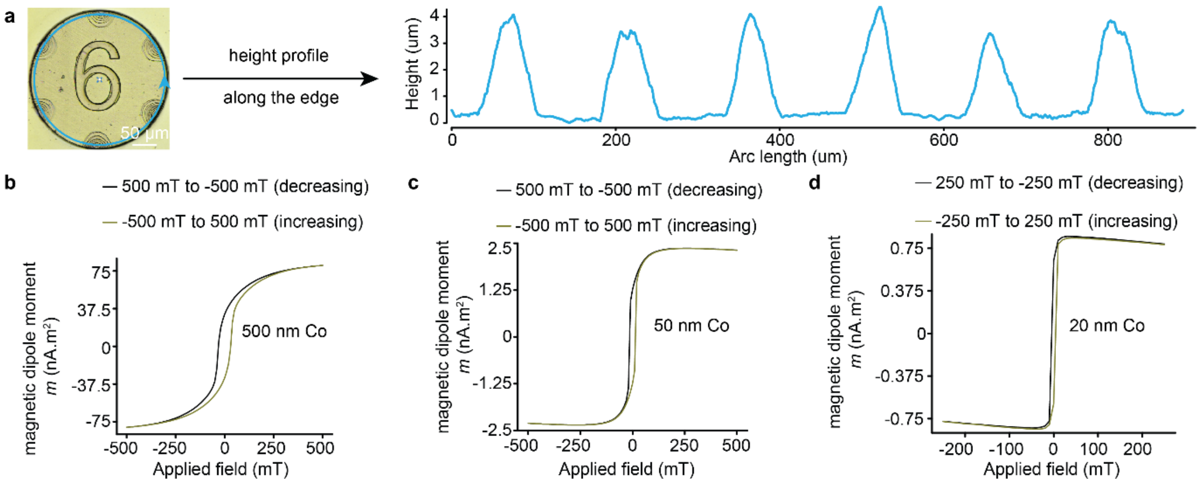

FIG. S1. Design and characterization of a micro-disk. (**a**) A laser confocal microscope image overlaid with optical image of a micro-disk and a plot of height profile along the edge of the disk. Scale bar is 50 μm. (**b-d**) Magnetic hysteresis curve for three samples with 500 nm-thick cobalt (b), 50 nm-thick cobalt (c) and 20 nm-thick cobalt (d). The hysteresis curves were measured for 10 mm x 10 mm glass piece and were scaled down to get the curves for a disk 300 μm in diameter.

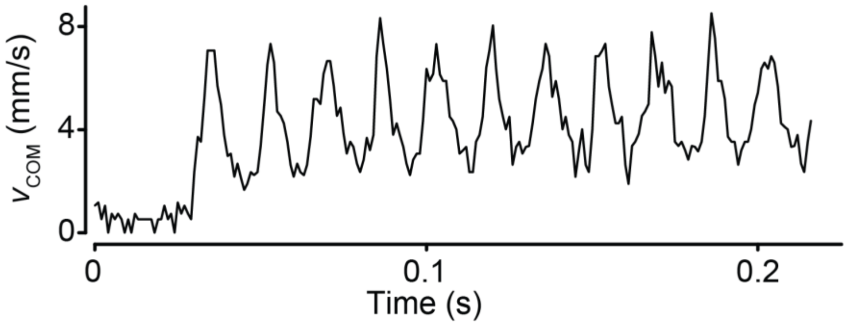

FIG. S2. Translation speed of the COM corresponding to the experiment shown in Figs. 1e and h.

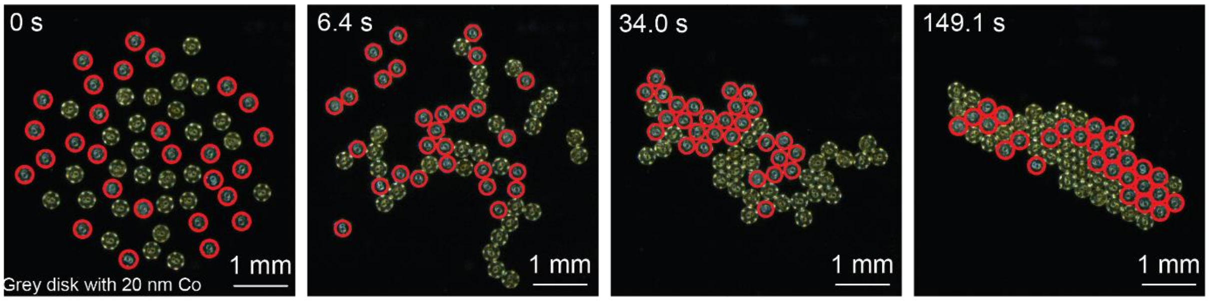

FIG. S3. Experimental images showing the tiling process of the heterogeneous collective. In this experiment, the rotation speed of the external magnetic field was 15 rotations per second (rps) for 5 s and then reduced to 1 rps for 15 s and then reduced to 0.25 rps for 10 s. Finally, a mix of two different rotating magnetic fields (Ω = 1 rps, B_0 = 3 mT for 1 s and Ω = 0.25 rps, B_0 = 0.5 mT for 4 s) was used for 120 s to break and minimize the number of holes in the tiles to form a single continuous tile. We observe that disks start to attach to each other at lower rotation speeds of the magnetic field (Ω < 10 rps). At lower rotation speed of the magnetic field the disks rotate slower, causing a decrease in the hydrodynamic repulsion between the disks. Therefore, the attractive interactions start dominating, causing the disks to assemble. At a very low rotation speed (Ω = 0.25 rps) the capillary interactions dominate and the six-fold symmetry in the capillary interactions causes the disks to assemble into hexatic-like tiles.

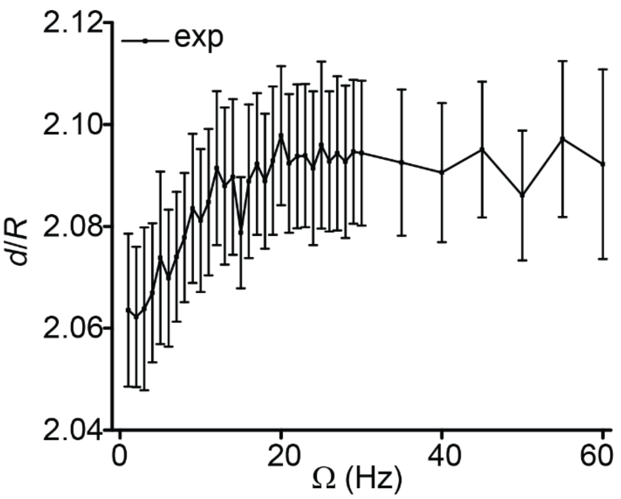

FIG. S4. Distance between the centers of a pair of type-1 and type-2 disks d vs frequency of the external magnetic field Ω. The distances are normalized by the radius of the disks R. The disks maintain a steady-state center-center distances that allow them to translate without needing any physical connection or external confinement. For each experiment, one value of Ω between 0 to 60 Hz was fixed and a video was recorded for 5 s at 30 frames per second. The distance between the disks was then extracted for each video frame using a custom python script.

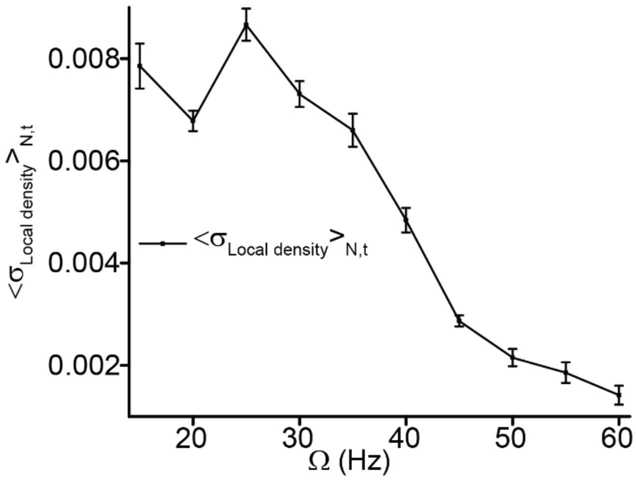

FIG. S5. Standard deviations of local density vs frequency of external magnetic field corresponding to the experiments shown in Fig. 3e, f, i.

Movie S1 (separate file). Behavior of a pair of micro-disks under 1D oscillating magnetic field. This Video is a compilation of representative experiments and simulations involving a pair of identical micro-disks under a uniform 1D oscillating magnetic field. The pair translates and rotates about its center of mass (COM) in the experiment. At the start of the movie, one of the disks changes its direction of rotation and as a result, both the disks rotate clockwise causing the pair to momentarily orbit clockwise about their common COM. This changes the direction of propulsion of the pair towards right. At around 13 s, the top disk starts to rotate counter-clockwise while the bottom disks still rotates clockwise and the pair propels towards right. At around 20 s, the bottom disk changes it's direction of rotation to counter-clockwise and both disks orbit counter-clockwise about their common COM for ~2 s. At around 23 s the bottom (now right) disk switches back to the clockwise rotation while the other disk still rotates counter-clockwise and the pair propels towards the top.

Movie S2 (separate file). Translation and rotation of a pair of micro-disks under 1D oscillating magnetic field. This video shows the translation and rotation of a pair of identical micro-disks under 1D oscillating magnetic field. The pair translate when both the disks have opposite directions of the instantaneous angular velocities and they orbit about a common center when both the disks have the same directions of the instantaneous angular velocities.

Movie S3 (separate file). Translation of a pair of magnetically non-identical micro-disks under 2D oscillating magnetic field. This video shows the experiments and simulations involving a pair of micro-disks with different magnetic moments. The type 1 (golden) micro-disk has stronger magnetic moment than the type 2 (grey) micro-disk. The pair translates along their axial direction. In the simulation video, a circle is drawn around the type 2 disk for visualization purpose.

Movie S4 (separate file). Steering a pair of magnetically non-identical micro-disks under 2D oscillating magnetic field. This video shows the experiments and simulations where the direction of translation a pair of micro-disks with different magnetic moment (type 1 and type 2) is steered using a 2D oscillating magnetic field. The mean axis of oscillation of the external magnetic field is changed from positive *Y*-axis to negative *X*-axis. In the simulation video, a circle is drawn around the type 2 disk for visualization purpose.

Movie S5 (separate file). Decoupling one type 1 disk (golden) from the rest of the collective and transporting it using a type 2 (grey) disk. This video shows the experiments where a single type 2 (grey) disk attaches to one type 1 (golden) disk and the pair gets decoupled from the rest of the collective. The experiment involved 31 type 1 (golden) disks and 1 type 2 (grey) disk. The type1 - type2 disk pair gets decoupled from the rest of the collective and it is shown to translate and steer along different directions. A red circle is added around the grey disk to distinguish between the two disk types.

Movie S6 (separate file). Many type 1 (golden) and type 2 (grey) disks behaving like a single homogeneous collective. This video shows experiments involving approximately 50 type 1 (golden) and 50 type 2 (grey) disks. The collective behaved like a homogeneous system under an external magnetic field rotating at 15 rps. Red circles are added around the grey disks to distinguish between the two disk types.

Movie S7 (separate file). Separation of type 1 (golden) and type 2 (grey) disks. This video shows experiments involving approximately 50 type 1 (golden) and 50 type 2 (grey) disks. The collective separated into groups of type 1 and type 2 disks and the type 2 disks get pushed towards the boundary. The external field rotates at 60 rps. Red circles are added around the grey disks to distinguish between the two disk types.

Movie S8 (separate file). Separation of type 1 (golden) and type 2 (grey) disks into rotating and assembled groups, respectively. This video shows experiments involving approximately 50 type 1 (golden) and 50 type 2 (grey) disks. The type 2 disks used in this experiment step-out at lower frequency (< 25 rps). Under an external rotating magnetic field, the collective separates into groups of type 1 and type 2 disks. The type 1 disks form a rotating group while the type 2 disks get assembled and get pushed out towards the edge of the rotating group. Red circles are added around the grey disks to distinguish between the two disk types.

Movie S9 (separate file). Subgroups of type 1 (golden) and type 2 (grey) disks translate in different directions. This video shows experiments involving approximately 50 type 1 (golden) and 50 type 2 (grey) disks. The collective breaks into subgroups under a 2D oscillating magnetic field. The subgroups with unequal number of type 1 and type 2 disks translate in different directions. Red circles are added around the grey disks to distinguish between the two disk types.

Movie S10 (separate file). Tiling of many type 1 (golden) and type 2 (grey) disks. This video shows the experiments involving tiling of many type 1 and type 2 disks. The angular speed of the magnetic field was 15 rotations per second (rps) for 5 s and then reduced to 1 rps for 15 s and to 0.25 rps for 10 s. Finally, a mix of two different rotating magnetic fields (Ω = 1 Hz, B_0 = 3 mT for 1 s and Ω = 0.25 Hz, B_0 = 0.5 mT for 4 s) was used to break the holes in the tilled structure. Red circles are added around the grey disks to distinguish between the two disk types.

Movie S11 (separate file). Type 1 (golden) disks exhibit various behaviors while type 2 disks (grey) stay assembled. This video shows a group of many type 1 and type 2 disks under different magnetic field profiles. The type 1 disks exhibit different formations (rotation, static, oscillation and gas-like mode) while the type 2 disks form an assembled group.

References

[1] W. Wang et al., *Order and Information in the Patterns of Spinning Magnetic Micro-Disks at the Air-Water Interface*, Sci. Adv. **8**, eabk0685 (2022).
